# Bulk Free Radical Terpolymerization of Butyl Acrylate, 2-Methylene-1,3-Dioxepane and Vinyl Acetate: Terpolymer Reactivity Ratio Estimation

**DOI:** 10.3390/polym16101330

**Published:** 2024-05-09

**Authors:** Maryam Movafagh, Kelly M. Meek, Alison J. Scott, Alexander Penlidis, Marc A. Dubé

**Affiliations:** 1 Department of Chemical and Biological Engineering, University of Ottawa, Ottawa, ON K1N 6N5, Canada; mmova103@uottawa.ca (M.M.); kelly.meek@concordia.ca (K.M.M.); 2 Department of Chemical and Materials Engineering, Concordia University, Montreal, QC H3G 1M8, Canada; 3 Department of Process Engineering and Applied Science, Dalhousie University, Halifax, NS B3H 4R2, Canada; alison.scott@dal.ca; 4 Institute for Polymer Research (IPR), Department of Chemical Engineering, University of Waterloo, Waterloo, ON N2L 3G1, Canada; penlidis@uwaterloo.ca

**Keywords:** reactivity ratios, terpolymerization, polymerization kinetics, 2-methylene-1,3-dioxepane (MDO), butyl acrylate, vinyl acetate

## Abstract

This investigation introduces the first estimation of ternary reactivity ratios for a butyl acrylate (BA), 2-methylene-1,3-dioxepane (MDO), and vinyl acetate (VAc) system at 50 °C, with an aim to develop biodegradable pressure-sensitive adhesives (PSAs). In this study, we applied the error-in-variables model (EVM) to estimate reactivity ratios. The ternary reactivity ratios were found to be r_12_ = 0.417, r_21_ = 0.071, r_13_ = 4.459, r_31_ = 0.198, r_23_ = 0.260, and r_32_ = 55.339 (BA/MDO/VAc 1/2/3), contrasting with their binary counterparts, which are significantly different, indicating the critical need for ternary system analysis to accurately model multicomponent polymerization systems. Through the application of a recast Alfrey–Goldfinger model, this investigation predicts the terpolymer’s instantaneous and cumulative compositions at various conversion levels, based on the ternary reactivity ratios. These predictions not only provide crucial insights into the incorporation of MDO across different initial feed compositions but also offer estimates of the final terpolymer compositions and distributions, underscoring their potential in designing compostable or degradable polymers.

## 1. Introduction

Since the early 1970s, there has been a growing awareness of the environmental challenges posed by plastic disposal, prompting discussions and initiatives to find viable solutions. In recent years, this issue has gained heightened urgency, extending beyond the realm of polymer science, and becoming a topic of widespread interest and debate [1]. Polymers, regarded as a fundamental human necessity, are prized for their lightweight nature, cost-effectiveness, and stability under diverse environmental conditions [2]. Although plastics play an integral role in modern industries, the majority are petroleum-derived and non-degradable, leading to indefinite lifetimes in landfills and elsewhere [3,4]. A key objective of the sustainable polymer community is to develop feedstocks and polymeric materials from renewable resources that can be easily degraded or chemically recycled [5,6].

A considerable portion of widely used polymers is derived from vinyl monomers, manufactured through free radical polymerization or other methods [7,8]. However, they often result in polymers with entirely carbon-based backbones, impeding efforts toward facile degradation. Radical ring-opening polymerization (rROP) has emerged as a valuable strategy, leveraging radical chemistry to induce the opening of cyclic monomers, thereby facilitating the introduction of degradable linkages into polymer backbones [9,10]. When combined with the radical polymerization of common olefinic comonomers, rROP permits the integration of degradable linkages into carbon–carbon backbones that would otherwise be nondegradable. The application of rROP copolymerization has predominantly centered around cyclic ketene acetals (CKAs) [11,12,13,14] with vinyl monomers.

The free radical polymerization of 2-methylene-1,3-dioxepane (MDO), a seven-membered CKA, enables the quantitative opening of its ring to yield an aliphatic polyester resembling poly(3-caprolactone) (PCL) [12,15], traditionally synthesized through metal-catalyzed ring-opening polymerization (ROP). Distinctions between PCL obtained via radical initiation and metal catalyst systems manifest in branching, crystallinity, and mechanical properties. Notably, the seven-membered CKAs exhibit greater steric hindrance of the non-ring-opened radicals and enhanced stability of the ring-opened radicals, collectively promoting a predominantly ring-opened structure in the final polymer. MDO can also be considered a bio-sourced monomer as it is produced from diols such as 1,4-butanediol and diethylene glycol, which are themselves bio-sourced [9].

Copolymerization stands as a pivotal process in tailoring the properties of polymer products, allowing for a meticulous adjustment of material characteristics through the manipulation of comonomer types and their incorporation in the resulting copolymer chains. The resulting tailored materials find applications in specific contexts, with the copolymer’s composition, composition distribution, molecular weight, and molecular weight distribution playing crucial roles in defining its properties [16]. When aiming to create (bio)degradable copolymers, it becomes imperative not only to incorporate sufficient degradable chemical linkages to produce short oligomers after degradation but also to ensure their regular distribution within and across chains [16,17,18]. Addressing the challenge of incorporating MDO units in acrylate-based polymers [18,19], specifically focusing on achieving a uniform distribution, a proposed solution entails estimating the reactivity ratios of the monomer system before initiating deeper studies in copolymerization and copolymer properties.

Reactivity ratios play a pivotal role in the kinetics of multicomponent polymerization systems. Despite the widespread use of terpolymerization in both industrial and academic settings, there remains a notable lack of studies dedicated to estimating reactivity ratios for such intricate systems. To effectively control copolymer or terpolymer composition, key parameters such as copolymer reactivity ratios (r_ij_) come into play. These ratios, expressing the ratio of the propagation rate constant of homopropagation to that of cross-propagation, allow predictions of polymerization rate, composition, sequence length, molecular weight, and, consequently, the performance characteristics of the final product. The Mayo–Lewis equation serves as a tool to calculate instantaneous polymer composition using these reactivity ratios [20]. A commonly accepted analogy exists between copolymerization and terpolymerization mechanisms, enabling researchers to apply reactivity ratios derived from binary pairs (obtained through copolymerization experiments) in models addressing terpolymerizations. Nevertheless, the direct application of binary reactivity ratios to terpolymerization systems is not universally applicable. Utilizing the binary–ternary analogy, even as an approximation, necessitates making significant assumptions about the system. When binary reactivity ratios are employed to characterize ternary systems, the potential outcomes encompass suboptimal performance in predicting terpolymer composition (not to mention molecular weight, polymerization rate, etc.), and inaccuracies in determining the characteristics of the resulting terpolymer product. In previous studies, the suggestion emerged that the direct estimation of ternary reactivity ratios from terpolymer composition data is preferable over the use of binary copolymer reactivity ratios [21,22]. Discrepancies in the prediction of terpolymer composition using binary reactivity ratios have been noted and are due to the presence of the third monomer [21] (more explanations on this are given later, below Equation (17)).

In this study, we focus on pressure-sensitive adhesives (PSAs), a class of polymers that exhibit adhesive properties with minimal pressure application. These adhesives form bonds upon contact with a surface and are extensively employed in applications where material adhesion and residue-free removal are paramount. The adhesive’s ability to balance elasticity and strength depends on factors such as the polymer’s glass transition temperature (*T_g_*). The *T_g_* characterizes the transition from a glassy (‘hard’, ‘solid-like’) to a ‘liquid-like’ (rubbery, flexible) state and is a critical determinant of adhesive performance. Achieving the desired *T_g_*, often below the application temperature necessitates a precise combination of monomers with varying *T_g_* values [23]. Monomers such as n-butyl acrylate (BA), 2-ethylhexyl acrylate (EHA), and methyl methacrylate (MMA) are frequently employed in PSA formulations (with homopolymer *T_g_* values of −54, −50, and 105 °C, respectively). Consequently, adhesive properties can be precisely controlled by manipulating the polymer composition through the combination of low *T_g_* monomers (i.e., BA and EHA) with the higher *T_g_* monomer (i.e., MMA) [24].

In this study, vinyl acetate (VAc) was chosen as the high *T_g_* monomer due to its well-documented favorable reactivity ratios with MDO, leading to random copolymerization, high conversion, and the generation of the ring-opened (ester) form of MDO [18,25,26,27,28,29]. Furthermore, for PSA applications, we specifically chose BA as the low *T_g_* monomer, as previously mentioned. MDO is a bio-sourced monomer, and it should be noted that while BA and VAc utilized in this study are petroleum-based, they can also be bio-sourced [30]. This work introduces an estimation of bulk terpolymer reactivity ratios for BA/MDO/VAc at 50 °C. To our knowledge, this investigation represents the first attempt to estimate ternary reactivity ratios for the BA/MDO/VAc system.

## 2. Materials and Methods

### 2.1. Materials

n-BA (≥99% purity), VAc (≥99% purity) and azobisisobutyronitrile (AIBN, 98% purity) were purchased from Sigma Aldrich (Oakville, ON, Canada). MDO was obtained from Wacker Chemie (München, Germany) and was used as received. Deuterated chloroform (Fisher (Ottawa, ON, Canada), 99.8%) was used for characterization. Reagent grade solvents (Fisher) for sample workup (e.g., acetone, methanol) were used as packaged. BA and VAc monomers underwent a rigorous purification process, passing through inhibitor removal columns acquired from Sigma Aldrich. This process effectively eliminated any traces of inhibitors, such as hydroquinone or monomethyl ether hydroquinone.

### 2.2. Experimental Method

The experimental setup included terpolymerizations of BA/MDO/VAc conducted in glass ampoules at a constant temperature of 50 °C. Three separate monomer compositions were used: ~80/10/10, 10/80/10 and 10/10/80 (*w*/*w*/*w*) BA/MDO/VAc. Approximately 5 g of each monomer mixture, containing 0.1 wt.% AIBN, were carefully transferred into glass ampoules with dimensions of 150–180 mm in length, a tube diameter of 10 mm, a thickness of ~1 mm, and a volume of approximately 12 mL.

The reaction mixtures underwent degassing through three or more cycles of a freeze–pump–thaw procedure. Subsequently, the ampoules were flame-sealed and then immersed in a temperature-controlled water bath maintained at 50 °C. The ampoules were removed from the water bath at various times to ensure a range of conversions. The reactions were terminated by quenching the ampoules in an ice bath. All the samples were characterized for conversion using standard gravimetric techniques. After adequate cooling, the ampoules and their contents were weighed, and the contents were poured into pre-weighed dishes containing acetone. The monomer was soluble in acetone, whereas the polymer precipitated. The monomer–acetone–polymer mixtures were allowed to soak for 12 h and subsequently agitated in a wrist-action shaker for 30 min. The liquid was decanted, and the resulting polymers were left to dry in a vacuum oven at 50 °C overnight. For higher conversion (solid) samples, a freezing and breaking method was employed to extract the samples. Each ampoule was submerged in liquid nitrogen to rapidly freeze its contents. Once frozen, the ampoules were cautiously removed from the liquid nitrogen and safely fractured, granting access to the polymer samples contained within. Each sample was placed in toluene until fully dissolved, followed by the addition of methanol to induce polymer precipitation. The resulting solvent mixture was decanted, and the polymer samples were dried as above. For all samples, the dried polymers were weighed to calculate the conversion. Each dried sample was analyzed for composition using ^1^H-NMR spectroscopy.

### 2.3. Characterization

^1^H-NMR peak assignments were established with references to copolymers of BA/VAc [31], VAc/MDO [32], and BA/MDO [16]. To enhance precision in the ^1^H-NMR peak assignments for the BA/MDO/VAc terpolymer, poly(MDO) and BA/VAc copolymers were synthesized. Subsequently, these polymers were characterized using ^1^H-NMR spectroscopy.

**Figure 1:**
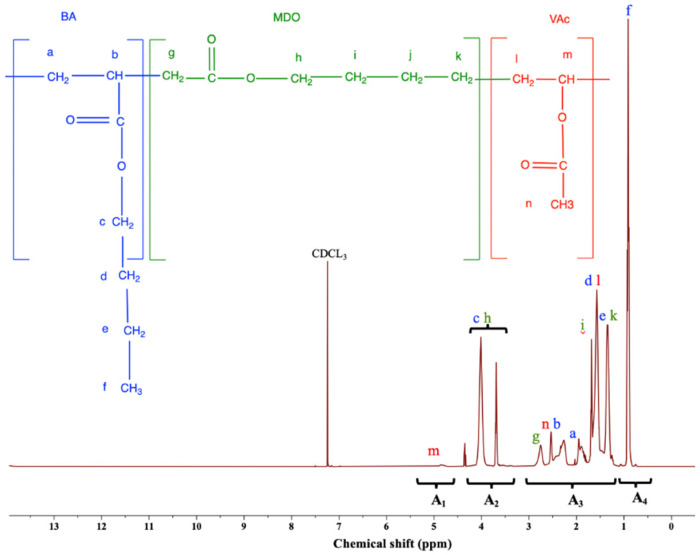
^1^H-NMR spectrum of a BA/MDO/VAc terpolymer with the feed composition of ~84/9/7 (mol%/mol%/mol%), respectively, at 82 wt.% conversion (F_BA_ = 74 mol%, F_MDO_ 20 mol%, F_VAc_ = 6 mol%). Blue: BA, green: MDO, red: VAc. Letters denote protons in molecular structure to peaks.

As previously mentioned, gravimetry was used to determine the monomer conversion based on the weight of the dry polymer relative to the weight of the starting reaction mixture. A Bruker 400 MHz ^1^H-NMR spectrometer was used to measure the compositions of the terpolymer. Samples were dissolved in deuterated chloroform (CDCl_3_) at a ratio of 0.02 g polymer to 1.5 g solvent. The spectrometer was configured for 1D analysis, generating 32 scans per minute. Figure 1 depicts ^1^H-NMR spectra for a typical BA/MDO/VAc terpolymer, along with peak assignments. The BA/MDO/VAc terpolymer compositions were calculated using:

A_1_ = z (1)

A_2_ = 2*x* + 2*y* (2)

A_3_ = 7*x* + 8*y* + 5*z* (3)

A_4_ = 3*x* (4)

*F*_BA_ = *x*/(*x + y + z*) (5)

*F*_MDO_ = *y*/(*x + y + z*) (6)

*F*_VAc_ = *z*/(*x + y + z*) (7)

where *x*, *y* and *z* relate to the BA, MDO and VAc protons, respectively, found in areas A_1_, A_2_, A_3_ and A_4_. Area A_1_ represents the −CH protons of VAc (“m” in Figure 1) (δ = 4.7–4.9 ppm), A_2_ represents the −CH_2_O protons of BA and MDO (“c” and “h” in Figure 1) (δ = 3.1–4.2 ppm), area A_4_ represents the −CH_3_ of BA (“f” in Figure 1) (δ = 0.7–1 ppm), whereas area A_3_ represents the remaining 20 protons of BA, MDO and VAc (δ = 1.1–2.9 ppm). After solving for *x*, *y*, and *z* (Equations (1)–(4)), the terpolymer composition was calculated using Equations (5)–(7). This composition analysis was conducted for all the BA/MDO/VAc terpolymers.

### 2.4. Reactivity Ratio Estimation in Terpolymerization

Terpolymerization systems, which involve the polymerization of three different monomers, indeed present a complex and rich area for research in polymer science. These systems are more complex than copolymerization, where only two monomers are involved, due to the larger number of possible interactions and resulting polymer structures. The increased complexity in terpolymerization arises from the various combinations in which the monomers can react, leading to a wide variety of polymer properties and applications.

**Scheme 1:**
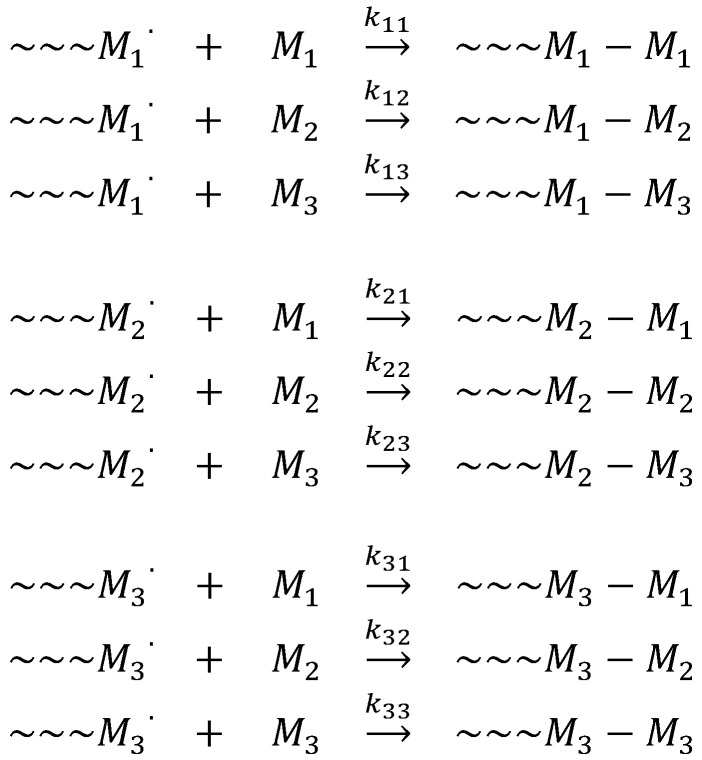
Terminal model for terpolymer propagation reactions.

The kinetics of terpolymerization systems, as first described by Alfrey and Goldfinger [33], consider the various possible interactions between the monomers. In a terpolymerization system, three different monomers can act as the terminal monomer on the growing polymer chain (Scheme 1). Thus, the growing polymer radical chain ending in monomer *i* (designated as ~~${M_{i}}^{\cdot }$) can react with any of the three monomers (*M_i_*) in the reaction mixture, leading to nine different propagation reaction (*k_ij_*) steps according to the terminal model [33]:

The propagation rate parameters, *k_ij_*, represent the rate of addition of monomer *j* to a growing radical chain ending in monomer *i*. To predict the terpolymer composition, one uses the reactivity ratios, denoted as $r_{ij}=\frac{k_{ii}}{k_{ij}}$, which indicates the propensity of a monomer to homopropagate (*k_ii_*), rather than cross-propagate (*k_ij_*). Thus, for a terpolymer system, six reactivity ratios can be defined [22,33]:

(8) $$\begin{array}{ccc} r_{12}=\frac{k_{11}}{k_{12}} & \;\;\;r_{13}=\frac{k_{11}}{k_{13}} & {\;\;r}_{23}=\frac{k_{22}}{k_{23}}\\ r_{21}=\frac{k_{22}}{k_{21}} & {\;\;r}_{31}=\frac{k_{33}}{k_{31}} & \;\;r_{32}=\frac{k_{33}}{k_{32}} \end{array}$$

The Alfrey–Goldfinger (AG) equations conventionally employ ratios of the instantaneous mole fractions within the terpolymer as the response variables (note: by instantaneous, we mean the terpolymer composition of the polymer chains generated at a single moment, as opposed to the cumulative composition, which is what is measured via ^1^H-NMR spectroscopy). However, in practice, experimental data often consist of individual mole fractions rather than their ratios. This leads to a loss of information (when using ratios) and alters the error structure when applying these ratios directly to experimental findings [22]. To address these limitations, the AG model was re-derived to allow each terpolymer mole fraction to be presented as a single response [22]. This re-derivation aligns with the need for a more accurate and practical approach to model terpolymerization kinetics, as it preserves the integrity of the experimental data and facilitates a more straightforward interpretation of results. The revised equations (Equations (9)–(11)) are designed to provide a more accurate representation of the terpolymer composition without the complications introduced by using ratio-based responses [22]. Therein, *F_i_* represents the instantaneous mole fraction of monomer *i* bound in the terpolymer, *f_i_* represents the mole fraction of unreacted monomer *i* in the polymerizing mixture, and the reactivity ratios (*r_ij_*) from Equation (8) are included.

(9) $$F_{1}-\frac{f_{1}\left(\frac{f_{1}}{r_{21}r_{31}}+\frac{f_{2}}{r_{21}r_{32}}+\frac{f_{3}}{r_{31}r_{23}}\right)\left(f_{1}+\frac{f_{2}}{r_{12}}+\frac{f_{3}}{r_{13}}\right)}{\left(\begin{array}{c} f_{1}\left(\frac{f_{1}}{r_{21}r_{31}}+\frac{f_{2}}{r_{21}r_{32}}+\frac{f_{3}}{r_{31}r_{23}}\right)\left(f_{1}+\frac{f_{2}}{r_{12}}+\frac{f_{3}}{r_{13}}\right)\\ +f_{2}\left(\frac{f_{1}}{r_{12}r_{31}}+\frac{f_{2}}{r_{12}r_{32}}+\frac{f_{3}}{r_{13}r_{32}}\right)\left(f_{2}+\frac{f_{1}}{r_{21}}+\frac{f_{3}}{r_{23}}\right)\\ +f_{3}\left(\frac{f_{1}}{r_{13}r_{21}}+\frac{f_{2}}{r_{23}r_{12}}+\frac{f_{3}}{r_{13}r_{23}}\right)\left(f_{3}+\frac{f_{1}}{r_{31}}+\frac{f_{2}}{r_{32}}\right) \end{array}\right)}=0$$

(10) $$F_{2}-\frac{f_{2}\left(\frac{f_{1}}{r_{12}r_{31}}+\frac{f_{2}}{r_{12}r_{32}}+\frac{f_{3}}{r_{13}r_{32}}\right)\left(f_{2}+\frac{f_{1}}{r_{21}}+\frac{f_{3}}{r_{23}}\right)}{\left(\begin{array}{c} f_{1}\left(\frac{f_{1}}{r_{21}r_{31}}+\frac{f_{2}}{r_{21}r_{32}}+\frac{f_{3}}{r_{31}r_{23}}\right)\left(f_{1}+\frac{f_{2}}{r_{12}}+\frac{f_{3}}{r_{13}}\right)\\ +f_{2}\left(\frac{f_{1}}{r_{12}r_{31}}+\frac{f_{2}}{r_{12}r_{32}}+\frac{f_{3}}{r_{13}r_{32}}\right)\left(f_{2}+\frac{f_{1}}{r_{21}}+\frac{f_{3}}{r_{23}}\right)\\ +f_{3}\left(\frac{f_{1}}{r_{13}r_{21}}+\frac{f_{2}}{r_{23}r_{12}}+\frac{f_{3}}{r_{13}r_{23}}\right)\left(f_{3}+\frac{f_{1}}{r_{31}}+\frac{f_{2}}{r_{32}}\right) \end{array}\right)}=0$$

(11) $$F_{3}-\frac{f_{3}\left(\frac{f_{1}}{r_{13}r_{21}}+\frac{f_{2}}{r_{23}r_{12}}+\frac{f_{3}}{r_{13}r_{23}}\right)\left(f_{3}+\frac{f_{1}}{r_{31}}+\frac{f_{2}}{r_{32}}\right)}{\left(\begin{array}{c} f_{1}\left(\frac{f_{1}}{r_{21}r_{31}}+\frac{f_{2}}{r_{21}r_{32}}+\frac{f_{3}}{r_{31}r_{23}}\right)\left(f_{1}+\frac{f_{2}}{r_{12}}+\frac{f_{3}}{r_{13}}\right)\\ +f_{2}\left(\frac{f_{1}}{r_{12}r_{31}}+\frac{f_{2}}{r_{12}r_{32}}+\frac{f_{3}}{r_{13}r_{32}}\right)\left(f_{2}+\frac{f_{1}}{r_{21}}+\frac{f_{3}}{r_{23}}\right)\\ +f_{3}\left(\frac{f_{1}}{r_{13}r_{21}}+\frac{f_{2}}{r_{23}r_{12}}+\frac{f_{3}}{r_{13}r_{23}}\right)\left(f_{3}+\frac{f_{1}}{r_{31}}+\frac{f_{2}}{r_{32}}\right) \end{array}\right)}=0$$

These equations were developed strictly for instantaneous composition measures. By utilizing data from low conversion processes, one assumes that changes in the composition of the terpolymer over time are minimal, meaning the overall cumulative composition measured can be closely equated to its instantaneous state at low conversions. Nonetheless, this limiting condition leads to potential errors for fast polymerizations and from composition drift from systems with widely differing reactivity ratios (e.g., BA/VAc [34]).

To address these challenges, a cumulative ternary composition model that considers the full conversion trajectory of the polymerization process was introduced [35]. This model, detailed in Equations (12)–(14), connects the cumulative composition of each comonomer in the terpolymer ($\bar{F_{i}}$) with the initial feed’s monomer mole fraction ($f_{i,0}$), the mole fraction of unreacted monomer within the polymerizing mixture (*f_i_*), and the total molar conversion (*X_n_*) [35].

(12) $$\bar{F_{1}}=\frac{f_{1,0}-f_{1}\left(1-X_{n}\right)}{X_{n}}$$

(13) $$\bar{F_{2}}=\frac{f_{2,0}-f_{2}\left(1-X_{n}\right)}{X_{n}}$$

(14) $$\bar{F_{3}}=\frac{f_{3,0}-f_{3}\left(1-X_{n}\right)}{X_{n}}$$

When a constant composition cannot be presumed, i.e., when composition drift becomes significant, *f_i_* needs to be assessed with respect to the conversion trajectory, as depicted in Equations (15)–(17).

(15) $$\frac{{df}_{1}}{dX_{n}}=\frac{f_{1}-F_{1}}{1-X_{n}}$$

(16) $$\frac{{df}_{2}}{dX_{n}}=\frac{f_{2}-F_{2}}{1-X_{n}}\;$$

(17) $$\frac{{df}_{3}}{dX_{n}}=\frac{f_{3}-F_{3}}{1-X_{n}}$$

This approach allows for a more accurate accounting of composition changes over time, particularly in systems where composition drift cannot be ignored [21]. The complexity of terpolymerization is underscored by the fact that the ternary reactivity ratios are not independent of each other and must be estimated simultaneously from the terpolymerization data, as their values can influence one another. For instance, despite the reactivity ratio between comonomers 1 and 2, *r*_12_ = $\frac{k_{11}}{k_{12}}$, which primarily reflects the propagation relation between comonomers 1 and 2, the presence of monomer 3 in the polymerization mixture can still exert an influence on it. Unlike copolymer systems, which involve two monomers, the addition of a third monomer can affect the polymerization behavior and the incorporation rates of all three monomers. Therefore, it is crucial to study and model terpolymerization processes on their own terms, rather than trying to extrapolate from simpler copolymer systems [35]. That way, one also avoids error propagation via binary copolymer reactivity ratios into the terpolymer composition. From a parameter estimation point of view, one obtains much richer information content due to the inclusion of conversion and cumulative terpolymer composition data. Last but not least, properly estimating binary copolymer reactivity ratios would require a minimum of twelve copolymerizations (with appropriate replication, which is often ignored), compared to only three terpolymerization runs, as long as the terpolymerization feed fractions are optimally located (hence, even with full independent replication, which is a very good feature to have, the number of trials is reduced by 50%).

Challenges associated with reactivity ratio estimation and experimental design in copolymer and terpolymer systems have largely been addressed through the implementation of the error-in-variables model (EVM), extensively discussed by Kazemi et al. [22]. The EVM technique stands out as a robust non-linear regression approach, encompassing all sources of experimental error in both the independent and dependent variables [20,21,36]. When utilizing EVM, the experimenter must carefully account for all sources of error, and the EVM procedure yields estimates of the true values of the independent variables within the model, alongside parameter estimates [21]. An additional advantage of employing EVM lies in its compatibility with the cumulative composition model for medium-high conversion data in terpolymer systems. This alternative offers several advantages over the standard instantaneous model, particularly in eliminating the assumption of negligible composition drift (required for the instantaneous model) and retaining more information content, i.e., more data points across the conversion trajectory, from a single experiment. Consequently, EVM emerges as the most statistically sound and comprehensive approach for reactivity ratio estimation [37]. The versatility of the EVM algorithm extends to its direct application to terpolymerization data, obviating the need for relying on binary reactivity ratios in ternary systems. Detailed procedures for this application have been previously clarified by Kazemi et al. [22]. The Direct Numerical Integration (DNI) method described therein can be applied to the ternary cumulative composition model, enabling the use of data up to medium-high conversion levels.

## 3. Results and Discussion

**Figure 2:**
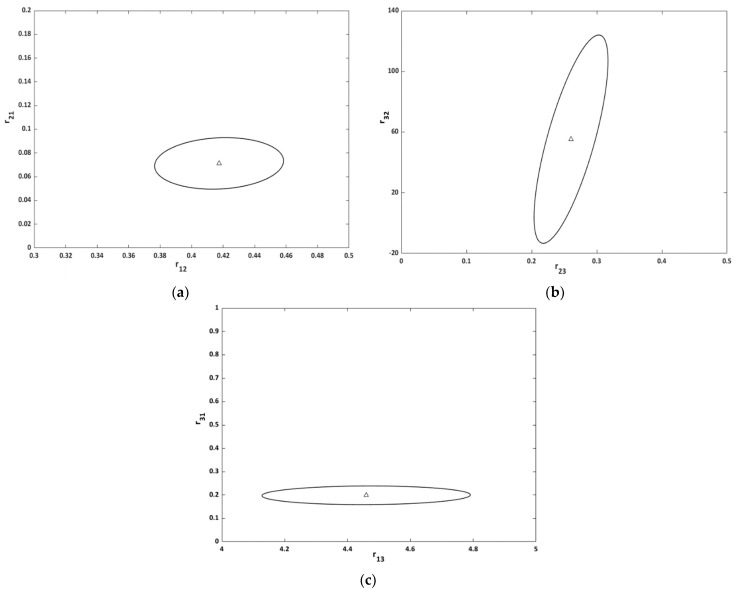
95% Joint Confidence Regions (JCRs) of the BA/MDO/VAc ternary reactivity ratio estimates (BA/MDO/VAc designated as monomers 1/2/3). Thus, (**a**) represents the BA/MDO reactivity ratios, (**b**) shows the MDO/VAc reactivity ratios, and (**c**) the BA/VAc reactivity ratios. JCRs are shown as lines, whereas point estimates as triangles.

This investigation utilized the EVM method to determine the reactivity ratios for BA/MDO/VAc terpolymerization (BA/MDO/VAc 1/2/3). Using a MATLAB-based EVM program [20,22], we analyzed the data, which included monomer feed composition (the manipulated variable), conversion (measured by gravimetry), and cumulative copolymer composition (measured by ^1^H-NMR spectroscopy). Table 1 shows the ternary reactivity ratios along with the copolymer reactivity ratios used as starting values in the EVM procedure (and related computer programs). The quality of the ternary reactivity ratio estimates was supported by clearly defined joint confidence regions (JCRs), shown in Figure 2. A direct comparison between the binary reactivity ratios, reported from three separate copolymer data sets in the literature, and the ternary reactivity ratios, obtained from a singular experimental data set, highlights substantial disparities (Table 1).

Figure 2 presents the ternary reactivity ratio point estimates within their 95% JCRs derived for each monomer pair. The JCRs serve as a quantitative measure of the uncertainty associated with the point estimates, offering insight into their reliability. The area encompassed by a JCR is inversely related to the precision of the corresponding point estimate: a smaller JCR area signifies lower variance and, consequently, higher reliability of the estimates. Based on our analysis, two interesting remarks can be made from the outset. First, the three JCRs of Figure 2 do not encompass the estimates from the binary reactivity ratios (this will be discussed further below). Secondly, almost all JCRs are parallel to one of the axes, thus indicating minimum covariance (correlation) between the respective parameters, which is a good feature, offering more confidence in the design of experiments.

For the BA/MDO pair (Figure 2a), the reactivity ratio point estimates r_12_ = 0.417 and r_21_ = 0.071 are both below 1, indicating that both BA and MDO tend to cross-propagate rather than homopolymerize. In other words, a BA-ended radical will more likely add an MDO monomer than a BA monomer. This tendency is much higher, however, for the MDO-ended radicals signifying that the generation of an uninterrupted series of MDO units in a chain is highly unlikely. The JCR in Figure 2a, shows a relatively equal degree of low uncertainty in each of the reactivity ratios. For the BA/VAc pair (Figure 2c), the reactivity ratio point estimates of r_13_ = 4.459 and r_31_ = 0.198 indicate the propensity of both BA- and VAc-ended radicals to add BA monomer rather than VAc monomer. This suggests a tendency to generate a relatively longer series of BA units in the terpolymer, i.e., BA “blockiness”. As was the case in Figure 2a, the JCR in Figure 2c shows again a relatively equal degree of low uncertainty in each of the reactivity ratios. Finally, for the MDO/VAc pair (Figure 2b), the reactivity ratio point estimates of r_23_ = 0.260 and r_32_ = 55.339 indicated the strong tendency for both MDO- and VAc-ended radicals to add VAc monomer rather than MDO monomer. While these reactivity ratios suggest that long sequences of VAc monomer units in the polymer chains would be likely, one cannot take these reactivity ratios in isolation from those of the other pairs. In other words, the presence of BA in the system and the BA/VAc reactivity ratios indicate the low likelihood of a series of VAc monomer units in the polymer chain. The JCR in Figure 2b shows a much higher uncertainty in r_32_ with relatively higher confidence in r_23_.

**Figure 3:**
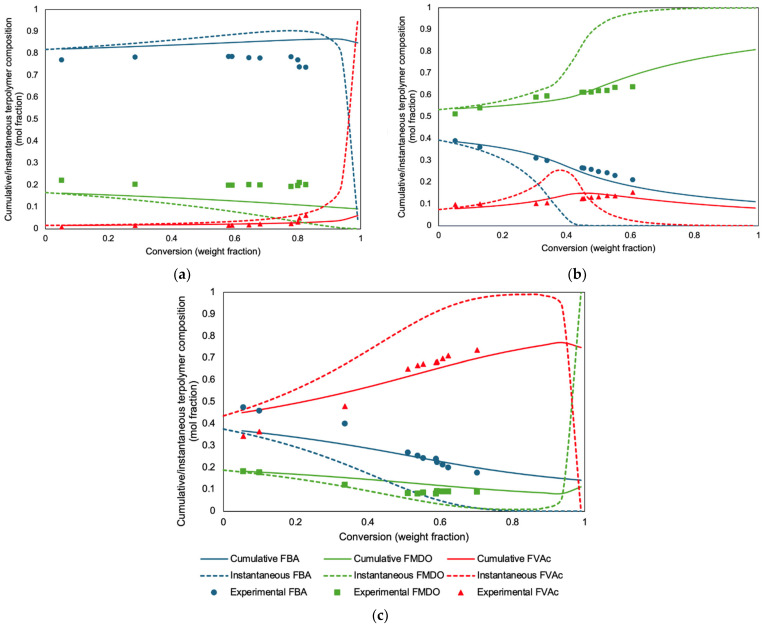
Comparison of model predictions of cumulative and instantaneous terpolymer compositions using ternary reactivity ratios and experimental data for BA/MDO/VAc terpolymerizations at various feed compositions (BA/MDO/VAc (mol%/mol%/mol%) (**a**) 84/9/7, (**b**) 11/81/8, and (**c**) 14/12/74). Experimental data are shown as symbols for BA (blue circles), MDO (green squares) and VAc (red triangles), colors of dashed and solid lines correspond to the same monomers.

**Figure 4:**
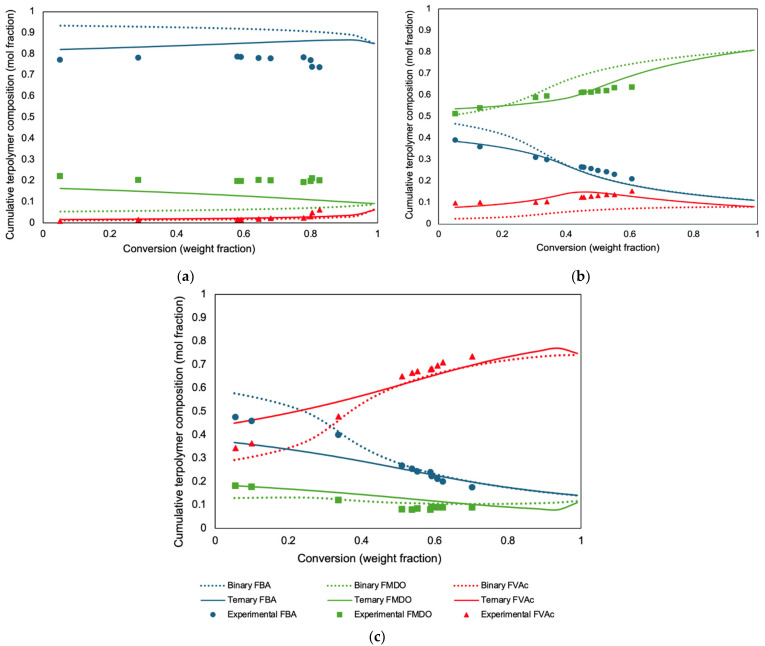
Comparison of model predictions of cumulative terpolymer composition using binary reactivity ratios (dotted lines) vs. using ternary reactivity ratios (solid lines) for BA/MDO/VAc terpolymerizations at various feed compositions (BA/MDO/VAc (mol%/mol%/mol%) (**a**) 84/9/7, (**b**) 11/81/8, and (**c**) 14/12/74). Experimental data are shown as symbols for BA (blue circles), MDO (green squares) and VAc (red triangles), colors of dashed and solid lines correspond to the same monomers.

In Figure 3, the agreement between the experimental data and the model predictions for the cumulative and instantaneous compositions of BA/MDO/VAc terpolymerizations using the ternary reactivity ratios is shown for the three compositions studied. Overall, the experimental data align well with the model predictions for cumulative compositions. Arguably, the predictions for the BA/MDO/VAc = 84/9/7 molar feed composition (Figure 3a) for BA and MDO are not perfect. The same holds for some of the low conversion data for BA and VAc for the BA/MDO/VAc = 14/12/74 molar feed composition (Figure 3c). However, these predictions are vastly better than those using the copolymer reactivity ratios (Figure 4). In general, the ternary composition predictions are consistent with the measured cumulative terpolymer compositions.

Recall that the motivation for this study was to produce adhesives with a reasonable distribution of MDO to ensure better degradability (or compostability). This compels us to consider how the instantaneous terpolymer composition behaves. The instantaneous terpolymer compositions shown in Figure 3 (an added benefit from mathematical modeling) all suggest significant composition drift for the BA/MDO/VAc system, particularly at higher conversions in Figure 3a, but even at fairly low conversions in Figure 3b,c. Thus, the composition drift was most notable for the MDO- and VAc-rich systems. For example, the significant composition drift in the MDO-rich system (Figure 3b), as noted through the instantaneous composition, shows that the production of polymer chains beyond 70% conversion would be dominated by MDO. The VAc-rich system (Figure 3c) exhibits a temporary halt in MDO incorporation between 75 and 85% conversion, followed by a resumed increase in MDO incorporation into the polymer. For both these cases, at higher conversion levels the BA monomer will have been almost completely converted to polymer, thus, the remaining monomer will be dominated by VAc and/or MDO. These two cases contrast with the BA-rich system (Figure 3a), where MDO continues to be incorporated into the polymer up to 95% conversion, suggesting a more uniform distribution of MDO throughout the polymerization process. In terms of compostability or degradability, one desires a uniform distribution of MDO and thus, the terpolymer composition in Figure 3a would be desirable.

The question remains: how much MDO is required in the formulation and how much composition drift can be tolerated? Even a minimal addition of 1 mol% MDO to the polymer backbone has been shown to initiate degradation, underscoring MDO’s role in enhancing degradability [28]. However, this alone does not ensure degradability. Overall degradation and degradation rate are influenced by many factors such as polymer structure, environmental conditions (pH, temperature, humidity), and polymer molecular weight [38,39]. The research literature suggests that adding 5–10 mol% MDO to the polymer backbone is a practical starting point for achieving biodegradation [28,29,40]. At the very least, a 5 mol% MDO addition appears to be a reliable threshold for developing degradable (or compostable) polymers because their insertion at that frequency would mean the presence of an MDO moiety to enable the breakup of high molecular weight polymer chains into lower molecular weight segments, which are more easily degraded. Thus, the terpolymer composition in Figure 3a satisfies this condition. Finally, for applications as a PSA, the BA-rich system also satisfies the need for a low *T_g_*, with a theoretical value of −47 °C calculated based on the Fox equation [32,41,42,43]. Thus, the low composition drift, favorable MDO incorporation and applicability as a PSA make the system in Figure 3a an obvious choice for our future work.

A detailed examination of the binary reactivity ratio predictions (Figure 4) reveals that the compositions in the BA-rich (Figure 4a) and MDO-rich (Figure 4b) systems were not accurately predicted, particularly when contrasted with the predictions made using ternary reactivity ratios for the same formulations. The prediction of the cumulative compositions of all three components in all three cases clearly illustrates the discrepancy between the use of binary and ternary reactivity ratios. As anticipated from previous discussions (herein and in the references), this outcome supports the use of reactivity ratios derived from ternary data rather than using the reactivity ratios derived from copolymerization experiments.

For the BA/MDO pair, the binary reactivity ratio r_12_ (1.761) was significantly higher than its ternary counterpart (0.417). This suggests an increased propensity for BA to add to a growing MDO chain in the presence of VAc, contrary to the binary system where BA had a higher tendency to propagate with itself. This shift indicates a stronger interaction between BA and MDO in the ternary system, possibly leading to a polymer with a more balanced incorporation of BA and MDO units. For the BA/VAc interaction, the observed decrease in the r_13_ value from 5.938 in the binary system to 4.459 in the ternary system indicates a small shift in BA’s behavior. Despite both values being significantly greater than 1, indicating a predominant preference of BA for self-addition, the decrease in the ternary system reactivity ratio suggests that BA’s relative tendency to add to VAc has increased in the presence of MDO. Simultaneously, the increase in the r_31_ value from 0.026 to 0.198, although still below 1, indicates a stronger, yet still limited, tendency for VAc to add to BA in the ternary system compared to the binary system. The most striking changes were observed in the MDO/VAc pair, where the r_23_ value dramatically decreased from 0.950 (binary) to 0.260 (ternary), whereas the r_32_ value surged from 1.71 (binary) to 55.339 (ternary). The observed decrease in the r_23_ value from 0.950 in the binary system to 0.260 in the ternary system does indeed suggest a significant shift in MDO’s behavior towards VAc. Initially, with an r_23_ value close to 1 in the binary system, MDO showed a nearly equal preference for adding to VAc as to itself, indicating a balanced reactivity between homo- and cross-propagation. However, the substantial reduction in this value in the ternary system suggests that MDO’s preference for adding to VAc over itself has increased markedly. This shift could lead to a terpolymer with more frequent sequences of VAc units directly linked to MDO. The dramatic increase in the r_32_ signifies a substantial change in VAc’s affinity towards MDO in the ternary system. All of the above comments suggest that, generally, the addition of a third monomer to a copolymer can have unexpected impacts in several directions, depending on the application.

**Figure 5:**
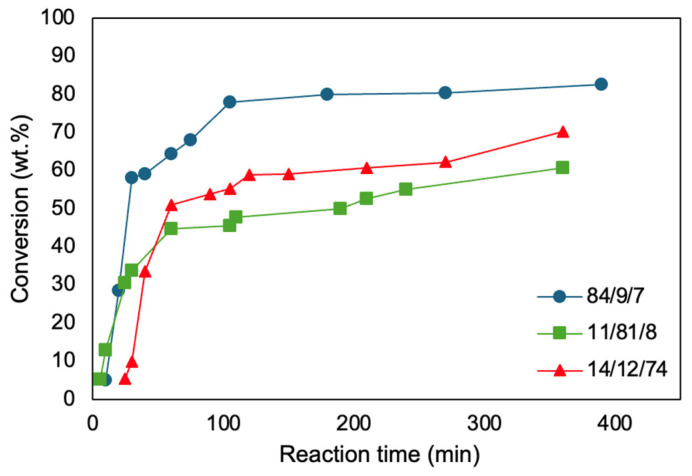
Conversion versus time for bulk BA/MDO/VAc terpolymerization at various feed compositions (BA/MDO/VAc (mol%/mol%/mol%) 84/9/7 (circles), 11/81/8 (squares), 14/12/74 (triangles)).

The monomer conversion versus time data for all three feed compositions are shown in Figure 5. The BA-rich system achieved the highest limiting conversion followed by the VAc-rich system and the MDO-rich system.

## 4. Conclusions

This study marks a significant advancement in the understanding of terpolymerization kinetics for the BA/MDO/VAc system, particularly through the accurate estimation of ternary reactivity ratios using EVM. Our findings reveal ternary reactivity ratios as r_12_ = 0.417, r_21_ = 0.071, r_13_ = 4.459, r_31_ = 0.198, r_23_ = 0.260, and r_32_ = 55.339 (BA/MDO/VAc as monomers 1/2/3), which contrast significantly with binary reactivity ratios, underscoring the complex nature of terpolymer systems and the necessity for ternary analysis. The application of a modified Alfrey–Goldfinger model enabled proper prediction of both instantaneous and cumulative terpolymer compositions, providing deep insight into the polymerization process and the specific incorporation behavior of MDO in varying feed compositions, in order to optimize formulations of degradable (compostable) polymer backbones.

These composition predictions are crucial for designing polymers with desired properties, particularly in the realm of biodegradable PSAs, where precise control over polymer composition and distribution is paramount. The study’s comprehensive approach offers a robust framework for accurately describing complex polymerization systems, paving the way for future innovations in sustainable polymer design.

## Figures and Tables

**Table 1:** Binary reactivity ratios (from literature) and ternary reactivity ratio estimates (BA/MDO/VAc designated as monomers 1/2/3).

Reactivity Ratios	r_12_	r_21_	r_13_	r_31_	r_23_	r_32_
Binary [16,34]	1.761	0.044	5.938	0.026	0.950	1.710
Ternary	0.417	0.071	4.459	0.198	0.260	55.339

## Data Availability

Data are contained withing the article.

## References

[1] Millican J.M., Agarwal S. (2021). Plastic Pollution: A Material Problem?. Macromolecules.

[2] Chamas A., Moon H., Zheng J., Qiu Y., Tabassum T., Jang J.H., Abu-Omar M., Scott S.L., Suh S. (2020). Degradation Rates of Plastics in the Environment. ACS Sustain. Chem. Eng..

[3] Schneiderman D.K., Hillmyer M.A. (2017). 50th Anniversary Perspective: There Is a Great Future in Sustainable Polymers. Macromolecules.

[4] Geyer R., Jambeck J.R., Law K.L. (2017). Production, Use, and Fate of All Plastics Ever Made. Sci. Adv..

[5] Dubé M.A., Salehpour S. (2014). Applying the Principles of Green Chemistry to Polymer Production Technology. Macromol. React. Eng..

[6] Dubé M.A., Gabriel V.A., Pakdel A.S., Zhang Y. (2021). Sustainable Polymer Reaction Engineering: Are We There Yet?. Can. J. Chem. Eng..

[7] Wan D., Satoh K., Kamigaito M., Okamoto Y. (2005). Xanthate-Mediated Radical Polymerization of N -Vinylpyrrolidone in Fluoroalcohols for Simultaneous Control of Molecular Weight and Tacticity. Macromolecules.

[8] Öztürk T., Savaş B., Meyvacı E., Kılıçlıoğlu A., Hazer B. (2020). Synthesis and Characterization of the Block Copolymers Using the Novel Bifunctional Initiator by RAFT and FRP Technics: Evaluation of the Primary Polymerization Parameters. J. Polym. Res..

[9] Folini J., Murad W., Mehner F., Meier W., Gaitzsch J. (2020). Updating Radical Ring-Opening Polymerisation of Cyclic Ketene Acetals from Synthesis to Degradation. Eur. Polym. J..

[10] Reddy Mothe S., Tan J.S.J., Chennamaneni L.R., Aidil F., Su Y., Kang H.C., Lim F.C.H., Thoniyot P. (2020). A Systematic Investigation of the Ring Size Effects on the Free Radical Ring-opening Polymerization (rROP) of Cyclic Ketene Acetal (CKA) Using Both Experimental and Theoretical Approach. J. Polym. Sci..

[11] Agarwal S. (2010). Chemistry, Chances and Limitations of the Radical Ring-Opening Polymerization of Cyclic Ketene Acetals for the Synthesis of Degradable Polyesters. Polym. Chem..

[12] Jin S., Gonsalves K.E. (1997). A Study of the Mechanism of the Free-Radical Ring-Opening Polymerization of 2-Methylene-1,3-Dioxepane. Macromolecules.

[13] Endo T. (2012). Radical Ring-Opening Polymerization. Polymer Science: A Comprehensive Reference.

[14] Tardy A., Nicolas J., Gigmes D., Lefay C., Guillaneuf Y. (2017). Radical Ring-Opening Polymerization: Scope, Limitations, and Application to (Bio)Degradable Materials. Chem. Rev..

[15] Bailey W.J., Ni Z., Wu S. (1982). Synthesis of Poly-ϵ-caprolactone via a Free Radical Mechanism. Free Radical Ring-opening Polymerization of 2-methylene-1,3-dioxepane. J. Polym. Sci. Polym. Chem. Ed..

[16] Lena J.-B., Jackson A.W., Chennamaneni L.R., Wong C.T., Lim F., Andriani Y., Thoniyot P., Van Herk A.M. (2020). Degradable Poly(Alkyl Acrylates) with Uniform Insertion of Ester Bonds, Comparing Batch and Semibatch Copolymerizations. Macromolecules.

[17] Weidner S., Kuehn G., Werthmann B., Schroeder H., Just U., Borowski R., Decker R., Schwarz B., Schmuecking I., Seifert I. (1997). A New Approach of Characterizing the Hydrolytic Degradation of Poly(Ethylene Terephthalate) by MALDI-MS. J. Polym. Sci. A Polym. Chem..

[18] Lena J.-B., Van Herk A.M. (2019). Toward Biodegradable Chain-Growth Polymers and Polymer Particles: Re-Evaluation of Reactivity Ratios in Copolymerization of Vinyl Monomers with Cyclic Ketene Acetal Using Nonlinear Regression with Proper Error Analysis. Ind. Eng. Chem. Res..

[19] Hill M.R., Guégain E., Tran J., Figg C.A., Turner A.C., Nicolas J., Sumerlin B.S. (2017). Radical Ring-Opening Copolymerization of Cyclic Ketene Acetals and Maleimides Affords Homogeneous Incorporation of Degradable Units. ACS Macro Lett..

[20] Polic A.L., Duever T.A., Penlidis A. (1998). Case Studies and Literature Review on the Estimation of Copolymerization Reactivity Ratios. J. Polym. Sci. A Polym. Chem..

[21] Scott A.J., Penlidis A. (2018). Binary vs. Ternary Reactivity Ratios: Appropriate Estimation Procedures with Terpolymerization Data. Eur. Polym. J..

[22] Kazemi N., Duever T.A., Penlidis A. (2014). Demystifying the Estimation of Reactivity Ratios for Terpolymerization Systems. AIChE J..

[23] Benedek I. (2004). Pressure-Sensitive Adhesives and Applications.

[24] Srivastava S. (2009). Co-Polymerization of Acrylates. Des. Monomers Polym..

[25] Wenzel F., Hamzehlou S., Pardo L., Aguirre M., Leiza J.R. (2021). Kinetics of Radical Ring Opening Polymerization of the Cyclic Ketene Acetal 2-Methylene-1,3-Dioxepane with Vinyl Monomers. Ind. Eng. Chem. Res..

[26] Hedir G., Stubbs C., Aston P., Dove A.P., Gibson M.I. (2017). Synthesis of Degradable Poly(Vinyl Alcohol) by Radical Ring-Opening Copolymerization and Ice Recrystallization Inhibition Activity. ACS Macro Lett..

[27] Bell C.A., Hedir G.G., O’Reilly R.K., Dove A.P. (2015). Controlling the Synthesis of Degradable Vinyl Polymers by Xanthate-Mediated Polymerization. Polym. Chem..

[28] Undin J., Illanes T., Finne-Wistrand A., Albertsson A.-C. (2012). Random Introduction of Degradable Linkages into Functional Vinyl Polymers by Radical Ring-Opening Polymerization, Tailored for Soft Tissue Engineering. Polym. Chem..

[29] Agarwal S., Kumar R., Kissel T., Reul R. (2009). Synthesis of Degradable Materials Based on Caprolactone and Vinyl Acetate Units Using Radical Chemistry. Polym. J..

[30] Niesbach A., Lutze P., Górak A. (2013). Reactive Distillation for Production of N-Butyl Acrylate from Bio-Based Raw Materials. Computer Aided Chemical Engineering.

[31] Abdollahi M., Massoumi B., Yousefi M.R., Ziaee F. (2012). Free-radical Homo- and Copolymerization of Vinyl Acetate and n -butyl Acrylate: Kinetic Studies by Online 1 H NMR Kinetic Experiments. J. Appl. Polym. Sci..

[32] Ding D., Pan X., Zhang Z., Li N., Zhu J., Zhu X. (2016). A Degradable Copolymer of 2-Methylene-1,3-Dioxepane and Vinyl Acetate by Photo-Induced Cobalt-Mediated Radical Polymerization. Polym. Chem..

[33] Alfrey T., Goldfinger G. (1944). Copolymerization of Systems of Three and More Components. J. Chem. Phys..

[34] Dubé M., Penlidis A. (1995). A Systematic Approach to the Study of Multicomponent Polymerization Kinetics? The Butyl Acrylate/Methyl Methacrylate/Vinyl Acetate Example: 1. Bulk Copolymerization. Polymer.

[35] Scott A.J., Gabriel V.A., Dubé M.A., Penlidis A. (2019). Making the Most of Parameter Estimation: Terpolymerization Troubleshooting Tips. Processes.

[36] Dubé M., Sanayei R.A., Penlidis A., O’Driscoll K.F., Reilly P.M. (1991). A Microcomputer Program for Estimation of Copolymerization Reactivity Ratios. J. Polym. Sci. A Polym. Chem..

[37] Scott A., Penlidis A. (2018). Computational Package for Copolymerization Reactivity Ratio Estimation: Improved Access to the Error-in-Variables-Model. Processes.

[38] Ghanbarzadeh B., Almasi H. (2013). Biodegradable Polymers. Biodegradation—Life of Science.

[39] Samir A., Ashour F.H., Hakim A.A.A., Bassyouni M. (2022). Recent Advances in Biodegradable Polymers for Sustainable Applications. NPJ Mater. Degrad..

[40] Lefay C., Guillaneuf Y. (2023). Recyclable/Degradable Materials via the Insertion of Labile/Cleavable Bonds Using a Comonomer Approach. Prog. Polym. Sci..

[41] Hagiopol C. (2005). Copolymers. Encyclopedia of Condensed Matter Physics.

[42] Fernandez-Garcia M., Cuervo-Rodriguez R., Madruga E.L. (1999). Glass Transition Temperatures of Butyl Acrylate-Methyl Methacrylate Copolymers. J. Polym. Sci. B. Polym. Phys..

[43] Browne E., Worku Z.A., Healy A.M. (2020). Physicochemical Properties of Poly-Vinyl Polymers and Their Influence on Ketoprofen Amorphous Solid Dispersion Performance: A Polymer Selection Case Study. Pharmaceutics.

